# Evaluating Swellable Cross-Linked Biopolymer Impact on Ink Rheology and Mechanical Properties of Drug-Contained 3D-Printed Thin Film

**DOI:** 10.3390/pharmaceutics17020183

**Published:** 2025-02-01

**Authors:** Farzana Khan Rony, Jonathan Appiah, Asmaa Alawbali, Distinee Clay, Shamsuddin Ilias, Mohammad A. Azad

**Affiliations:** 1 Department of Applied Science and Technology, North Carolina A&T State University, Greensboro, NC 27411, USA; fkrony@aggies.ncat.edu; 2 Materials Science and Process Engineering (MSPE) Lab, Department of Chemical, Biological, and Bioengineering, North Carolina A&T State University, Greensboro, NC 27411, USA; jappiah@aggies.ncat.edu (J.A.); djclay@aggies.ncat.edu (D.C.); 3 Department of Chemical, Biological, and Bioengineering, North Carolina A&T State University, Greensboro, NC 27411, USA; ilias@ncat.edu

**Keywords:** 3D printing, oral thin film, rheological properties, swellable cross-linked biopolymer, sodium starch glycolate (SSG), mechanical properties

## Abstract

**Background/Objectives:** Interest in 3D printing oral thin films (OTFs) has increased substantially. The challenge of 3D printing is film printability, which is strongly affected by the rheological properties of the ink and having suitable mechanical properties. This research assesses the suitability of sodium starch glycolate (SSG), a swellable cross-linked biopolymer, on ink rheology and the film’s mechanical properties. **Methods:** A water-based ink comprising sodium alginate (SA), the drug fenofibrate (FNB), SSG, glycerin, and polyvinylpyrrolidone (PVP) was formulated, and its rheology was assessed through flow, amplitude sweeps, and thixotropy tests. Films (10 mm × 15 mm × 0.35 mm) were 3D-printed using a 410 µm nozzle, 50% infill density, 60 kPa pressure, and 10 mm/s speed, with mechanical properties (Young’s modulus, tensile strength, and elongation at break) analyzed using a TA-XT Plus C texture analyzer. **Results:** The rheology showed SSG-based ink has suitable properties (shear-thinning behavior, high viscosity, higher modulus, and quick recovery) for 3D printing. SSG enhanced the rheology (viscosity and modulus) of ink but not the mechanical properties of film. XRD and DSC confirmed preserved FNB crystallinity without polymorphic changes. SEM images showed surface morphology and particle distribution across the film. The film demonstrated a drug loading of 44.28% (RSD 5.62%) and a dissolution rate of ~77% within 30 min. **Conclusions:** SSG improves ink rheology, makes it compatible with 3D printing, and enhances drug dissolution (formulation F-5). Plasticizer glycerin is essential with SSG to achieve the film’s required mechanical properties. The study confirms SSG’s suitability for 3D printing of OTFs.

## 1. Introduction

Oral thin films (OTFs) have emerged as a promising and prominent dosage form in the pharmaceutical industry, traditionally dominated by tablets and capsules [1]. OTFs are particularly beneficial for patients with swallowing difficulties, such as children, the elderly, and those with dysphagia [2,3]. OTFs also provide other advantages, including more accurate dosing, enhanced oral absorption, improved bioavailability through minimization of drug degradation, better stability, dose tunability, portability, etc. [4]. Three-dimensional (3D) printing has become increasingly important in drug delivery since it offers a safer and more effective method for administering potent therapeutics [5,6]. Recent advancements have expanded 3D printing in oral drug delivery, leading to innovative pharmaceutical dosage forms, including OTFs [7]. 3D printing presents a promising alternative for developing and manufacturing OTFs, overcoming the limitations of traditional techniques such as solvent casting or hot-melt extrusion. Unlike solvent casting, 3D printing allows for more customization of solid dosage forms for end-stage personalization [8,9]. 

Extrusion-based 3D printing, such as fused deposition modeling (FDM) and pressure-assisted microsyringe (PAM), has been widely studied in pharmaceutical manufacturing, including the development of OTFs [10,11,12,13]. These techniques construct 3D structures with precise composition and architecture by depositing materials layer by layer under the control of a computer [13]. Furthermore, these techniques are cost-effective and flexible when using various polymers, with or without drugs. Moreover, extrusion-based 3D printing helps control drug release by adjusting the geometry and polymer of the film [14]. In FDM 3D printing, the thermoplastic filament is melted or softened, then extruded and deposited layer by layer to form a 3D object [13]. In PAM printing, semi-solids like gels or pastes are continuously extruded through a syringe-based print head, typically using pneumatic, mechanical, or solenoid pistons [10,15]. PAM is ideal for thermally sensitive drugs and polypills and offers higher drug loading than FDM [16,17]. Although PAM does not require high temperatures like FDM, post-processing drying is necessary after printing, which can cause shrinking or deformation. The printed object may also collapse if the layers do not solidify enough to support the weight of subsequent layers [18]. Additionally, the rectilinear, hexagonal, or honeycomb infill pattern affects the printed structure’s mechanical strength (i.e., flexural strength) in 3D printing [10].

The key challenge with PAM 3D printing is developing a polymer-based ink with the appropriate rheological properties to ensure smooth extrusion through the print head and maintain its shape after deposition [12]. Inadequate rheological properties can lead to nozzle clogging or defective prints [19]. Proper printing parameters, tailored to the ink’s rheology, are also essential for achieving good printability and reproducibility [20]. Thus, it is crucial to formulate a polymer-based ink tailored for PAM-type 3D printing. Semi-solids require an optimal mixture of polymer, solvent, and other excipients to achieve the necessary rheological properties for successful printing [10]. The polymers can be natural or synthetic, with natural polymers being the most prevalent in the biomedical and pharmaceutical industries [21,22]. 

Sodium alginate (SA), a sodium salt of an anionic linear polysaccharide composed of β-d-mannuronic acid and its epimer α-l-guluronic acid, is widely used in the pharmaceutical industry and is generally recognized as safe (GRAS) [23,24]. Recent studies have explored the use of sodium alginate in ink formulations to see if it is suitable for extrusion-based 3D printing [25,26,27,28]. SA films typically lack the desirable mechanical properties [29]. To improve these mechanical properties, plasticizers are added to the polymer mix. A plasticizer is a small, miscible molecule that interacts with the film-forming polymer, moderates the polymer-polymer interactions, and increases polymer chain mobility [9]. This leads to a lower glass transition temperature, reduced tensile strength, and enhanced film flexibility [30]. Thus, plasticizers are crucial in formulating film-based drug delivery systems, as they increase polymer flexibility by reducing intermolecular forces, resulting in better patient compliance and adherence to pharmacotherapy mobility [9]. An effective plasticizer must be compatible with the drugs, solvents, and polymers. Commonly used plasticizers include sorbitol, mannitol, glycerin, diethyl phthalate, triethyl citrate, tributyl citrate, macrogol, propylene glycol, and citric acid esters, whereas glycerin is the most used plasticizer for alginate films [2,31].

Another key excipient in OTF formulations is swellable cross-linked biopolymers known as disintegrants, which promote disintegration through the synergistic action of water absorption and swelling [32]. Swellable disintegrant agents speed up the process by absorbing water and swelling, thus enhancing the bioavailability and disintegration properties of OTF. Common swellable disintegrants are sodium starch glycolate (SSG), croscarmellose sodium (CCS), and crospovidone (CP) [2]. Notably, SSG stands out for its ability to uptake water rapidly, followed by fast and massive swelling while remaining insoluble in water [33]. The SSG swellable disintegrant outperforms traditional high molecular weight viscosity agents like guar gum, xanthan gum, and pectin in improving film drug content uniformity, especially for poorly water-soluble drugs such as griseofulvin, fenofibrate, and fexofenadine [34,35,36]. 

It is also noted that SSG is a superior dispersant compared to traditional dispersants such as sugar (sucrose) and sugar alcohols (mannitol, sorbitol) [37]. A significantly lower amount of the SSG compared to traditional dispersants provides a similar range of rheological properties of ink suitable for 3D printing [38,39]. In addition to its lower quantity utilization compared to typical dispersants, SSG’s natural origin, biodegradability, and compatibility with pharmaceutical applications support the industry’s transition to more sustainable, environmentally friendly, and effective drug delivery methods [40,41]. A few articles have used SSG as a swellable disintegrator and viscosity enhancer of polymer paste formulations [32,34,35,42]. However, there is currently no research on how the addition of SSG affects the rheological properties of SA polymer inks, its role in 3D printing thin films, and its impact on the mechanical properties of films. 

This study explores how incorporating SSG, a swellable crosslinked biopolymer, into SA film-forming polymer ink enhances the ink’s rheological properties, such as viscosity, shear-thinning behavior, and viscoelasticity, leading to successful 3D printing using a PAM printer with accurate shape fidelity. We also assess how SSG influences the film’s mechanical properties and further improve these properties by adding glycerin as a plasticizer. Glycerin is used as a plasticizer because it is widely used and commonly found in pharmaceutical formulations [7,43,44]. Fenofibrate (FNB), which is used to treat high cholesterol levels, was used as a model drug in this study. 

## 2. Materials and Methods

### 2.1. Materials 

Alginic acid sodium salt (SA) was purchased from Acros Organics (Morris Plains, NJ, USA). Fenofibrate and polyvinylpyrrolidone K 30 (PVP K30) were purchased from Tokyo Chemical Industry Co., Ltd. (Tokyo, Japan). Cross-linked biopolymer sodium starch glycolate (Explotab) was donated by JRS PHARMA GmbH & Co. Kg (Rosenberg, Germany). As-received SSG was sieved using mesh openings of 75, 63, 45, and 25 µm nominal size. Particles smaller than 45 µm were used in this study. The sieving was performed to minimize clogging of the nozzle of the printing head because SSG particles swell significantly in water [45]. Polyethylene glycol 6000 (PEG 6000) and D-mannitol 97%+ were purchased from Alfa Aesar (Thermo Fisher Scientific, Ward Hill, MA, USA). Refined glycerin (99.7% vegetable grade) was purchased from Twin Rivers Technologies (Quincy, MA, USA).

### 2.2. Design of Thin Film

**Figure 1:**
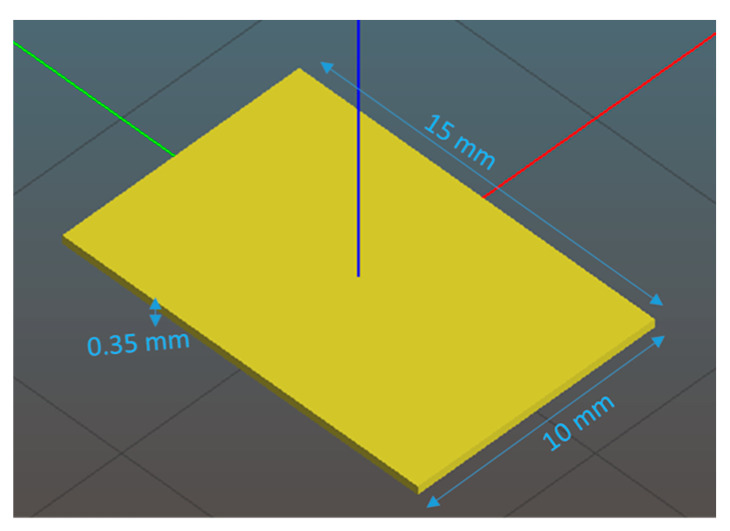
Design of 3D thin film using a CAD modeling program.

The printing process begins with the designing of a 3D structure in a CAD modeling program [19]. This 3D CAD file is converted into G-code, instructing the 3D printer head on movement, timing, and extrusion. A 3D slicing software (i.e., Slic3r) generates the G-code from the 3D CAD design. Finally, the printer constructs the structure according to the G-code instructions. In this study, Solidworks (Dassault Systèmes, 2018), a CAD program, was used to create the 3D model of the film (see Figure 1). The preliminary design for the film has dimensions of 15 mm in length, 10 mm in height, and 0.35 mm in thickness.

### 2.3. Preparation of Ink for Printing 

A total of 30 g batch-size printing ink was prepared using SA polymer with other excipients. A summary of the printing ink compositions studied is shown in Table 1. The ink F-1 was developed in the lab for pill 3D printing [19] and was later used for film printing. Despite high-quality prints, the resulting films were brittle and lacked the desirable mechanical (i.e., plasticizing) properties. Therefore, it became necessary to modify the ink formulations to ensure the 3D-printed films had suitable mechanical properties. Formulations F-2 through F-5’ were developed to enhance the ink’s rheological properties and the film’s plasticizing properties. The ink formulation F-1, containing 0.8% SA, was developed by grinding 0.24 g sodium alginate powder, 6 g fenofibrate, 7.5 g mannitol (filler), 3 g PEG (plasticizer), and 1.5 g PVP (stabilizer) with a mortar and pestle for 5 min. Afterward, 11.76 g of deionized (DI) water was added, and the mixture was processed (mixed, dispersed, and degassed) in a THINKY mixer (ARE–310) at 2000 rpm for 10 min, resulting in a uniform ink. For the preparation of ink formulations F-2 through F-5, the same procedure as F-1 was followed, with the only difference being that glycerin was added to the water before mixing with the dry ingredients in the THINKY mixer. For formulation F-5’, the dry ingredients were ground first, then added to the water and mixed using the THINKY mixer. 

It is noted that each ink formulation was modified by varying one parameter at a time to understand its impact on ink rheology and, eventually, the mechanical properties of successfully 3D-printed films. For example, formulations F-1 and F-2 were studied to evaluate the effect of plasticizers. Hence, formulation F-1 utilized PEG, whereas F-2 utilized glycerin as a plasticizer. All other materials and their compositions were kept the same. Formulation F-2’ was prepared to evaluate the rheology of the ink without mannitol. Formulation F-3 was prepared using SSG as a substitute for mannitol to enhance rheological properties suitable for 3D printing. Formulation F-4 involved increasing the SA polymer quantity to achieve optimal rheology for 3D printing. Formulation F-5 adjusted the viscosity of the ink for 3D printing by reducing the SSG quantity, resulting in favorable rheological properties. Finally, formulation F-5’ was developed to investigate the effect of SSG on the film’s mechanical (plasticizing) properties, with glycerin removed from the formulation. 

### 2.4. Fabrication of 3D Films

**Figure 2:**
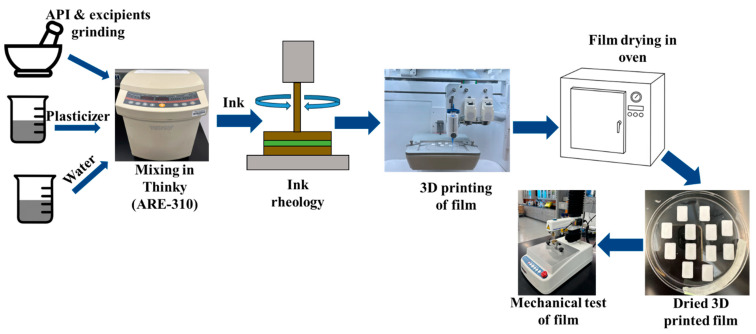
Schematic presentation of ink formulation and rheology, film 3D printing process, and subsequent mechanical testing.

**Figure 3:**
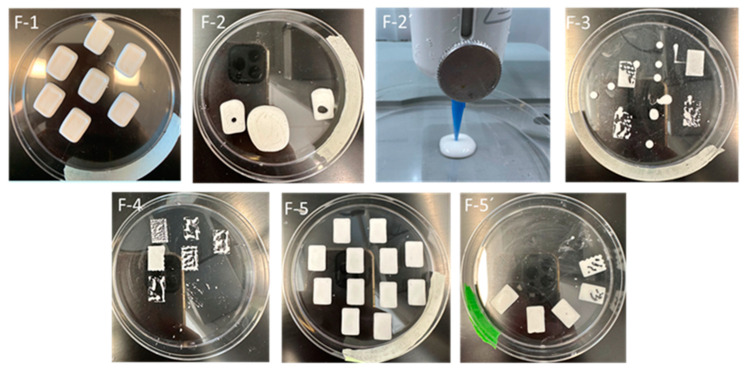
3D printing outcomes using different ink formulations for F-1, F-2, F-2’, F-3, F-4, F-5, and F-5’.

3D films were produced using each ink and the Cellink BioX 3D printer with the following procedure and printer settings: the prepared ink was loaded into a 3 mL cartridge and placed into the printing head. Then, a 410 μm nozzle was attached to the cartridge and positioned in the printer’s printhead. Printing was performed on 100 mm diameter plastic Petri dishes at a printing speed of 10 mm/s, an infill density of 50%, and a printing pressure of 60 kPa. The successfully printed films were dried at 40 °C for 24 hours (for F-1) and 48 hours (for F-5 and F-5’) in an oven (Heratherm OMS60, Thermo Fischer Scientific, Waltham, MA, USA) to evaporate any excess moisture. Films (F-5 and F-5’) containing glycerin required extended drying time. A short drying time results in inadequate water removal and sticky 3D-printed products [46]. It is noted that in this study, an optimization of drying was not performed. Figure 2 illustrates the ink formulation and rheology, the 3D film printing, and subsequent mechanical testing. 3D printing outcomes using different inks are presented in Figure 3.

### 2.5. Rheological Characterization of Ink

Rheology is the science of how a material flows and deforms when acted upon by a force [47]. The rheology of each ink was analyzed using an Anton Paar modular compact rheometer (MCR 302) with a PP measuring system, a Peltier hood, and a 25 mm parallel plate with a geometric gap of 1 mm. Flow, amplitude sweep, and thixotropy tests were investigated using the rheometer. All rheological tests were performed at 25 °C and a frequency of 1 Hz. Flow tests for each ink were performed with a 0.1 to 1000 1/s shear rate. The viscosity curves were generated by plotting the viscosity (mPa.s) vs. the shear rate (1/s). Amplitude sweep tests for each ink were performed with a shear strain range of 0.001 to 1000%. Thixotropy tests, consisting of three intervals, were conducted to measure the recoverable characteristics of the inks [48]. The first and third intervals involved oscillation at a shear strain percentage within the linear viscoelastic (LVE) region limit, determined from the amplitude sweep test, lasting 60 seconds and 145 seconds, respectively. The second interval involved applying a rotational shear rate of 100 1/s for 5 s to deform the sample.

### 2.6. Characterization Techniques of Film

#### 2.6.1. Mechanical Properties of Film

The film’s mechanical properties were evaluated following the procedure described in [49]. Measurements were taken using a TA-XT Plus C texture analyzer (Stable Microsystems, Godalming, UK). First, the film’s thickness was measured at three different locations using a digital caliper (World Precision Instruments, Sarasota, FL, USA), and the average thickness was used. The film (F-5) samples were fastened between two grips, with the lower grip remaining stationary while the upper grip moved at a test speed of 1 mm/s until the film broke. To evaluate the mechanical properties of the film, Young’s Modulus (YM), tensile strength (TS), and elongation at break (EB) were computed. YM was calculated as the slope of the linear portion of the stress–strain curve and expressed in force per unit area (kPa). TS was determined by dividing the tensile force at the film break by the film’s cross-sectional area, expressed in force per unit area (kPa). EB was calculated by dividing the extension at the moment of the film’s rupture by the initial length of the sample and multiplying this number by 100 to express it as a percentage (%). The average and the standard deviation were recorded from six replicates.

#### 2.6.2. X-Ray Diffractometer (XRD) Analysis

The x-ray diffraction patterns of the as-received FNB powder and the FNB-containing 3D film were measured using a Rigaku MiniFlex 600 benchtop XRD equipped with a scintillation counter detector. A monochromatic Cu Kα radiation source was used at an operating voltage and current of 40.0 kV and 15 mA, respectively. All samples were scanned over a 2θ range from 5 to 35° with a step size of 0.02° and a scanning rate of 2°/min speed.

#### 2.6.3. Differential Scanning Calorimetry (DSC)

A DSC analyzer (DSC 3^+^ with STAR^e^ system, Mettler-Toledo, Columbus, OH, USA) was used to determine the melting temperature of FNB, both as received powder and in the film. Approximately 1–5 mg of each sample was placed in a sealed, perforated aluminum pan of 40 µL capacity and loaded into the DSC. The samples were heated at a rate of 10 °C/min over a temperature range of 25–110 °C. Nitrogen gas was used as the purge gas. Data analysis was performed using STAR^e^ software (https://starsoftware.co/) provided by Mettler-Toledo.

#### 2.6.4. Scanning Electron Microscopy (SEM)

Morphologies of the 3D-printed films were investigated using scanning electron microscopy (SEM) (JSM-IT800, JEOL, Tokyo, Japan) with an accelerating voltage of 500 V and a current of 2 A. Before SEM, the film samples were double coated with approximately 14 nm of gold by a sputter coater (LEICA EM ACE 200). 

#### 2.6.5. Determination of Drug Loading and Uniformity in Films

The drug loading and uniformity in the films were evaluated by established protocols [49,50]. Six films (F-5) were dissolved in 250 mL of a 7.2 mg/mL SDS solution that was continuously stirred for 3 hours. The concentration of FNB in each dissolved sample was measured using a UV–vis spectrophotometer (Evolution 201 UV–visible Spectrophotometer, Thermo Fisher Scientific) by recording the absorbance at 286 nm wavelength [51]. A water-SDS solution (without drug) with a 7.2 mg/mL concentration was used as a blank control. The concentration of the FNB drug was calculated using the previously constructed calibration curve. The calibration curve was constructed by plotting absorbance versus known concentrations of FNB (0.8–25.11 mg/L) (Figure S1). The averages and relative standard deviation (RSD) of the weight percentage of FNB in the film were calculated from the six replicates. 

#### 2.6.6. Dissolution of Film

The dissolution of the films was investigated using a dissolution apparatus (Vision^®^ G2 Classic 6^TM^) equipped with 40-mesh 316 SS baskets following USP 1 [52]. Films were horizontally positioned in the basket, operated with a rotational speed of 50 rpm, and maintained at a temperature of 37 °C. A dissolution medium of 1000 mL [53] of 7.2 mg/mL SDS solution was used and maintained the sink condition [54]. Aliquots were withdrawn at 5, 10, 20, 30, 60, 90, and 120 min and immediately filtered using a 0.2 µm membrane filter (PVDF Filter Membrane with Polypropylene Housing). FNB concentration was evaluated by using a UV–visible spectrophotometer (Evolution 201 UV–Visible Spectrophotometer, Thermo Fisher Scientific) at 286 nm, and the same calibration curve was developed in Section 2.6.5. As a control, FNB as-received powder (40 mg) was also tested following the aforementioned procedure. The FNB as received powder was directly added into the dissolution vessel containing 1000 mL of 7.2 mg/mL SDS solution. Four replicates were recorded, and the average FNB release and standard deviation were plotted as a function of time. 

## 3. Results and Discussion

### 3.1. Evaluate Ink Rheology

The rheological properties of all the ink formulations (F-1–F-5’) were analyzed, compared, and evaluated to see if the formulations were suitable for 3D printing. A comprehensive evaluation of ink rheology for the formulations (F-1–F-5’) is discussed here.

#### 3.1.1. Flow Test

**Figure 4:**
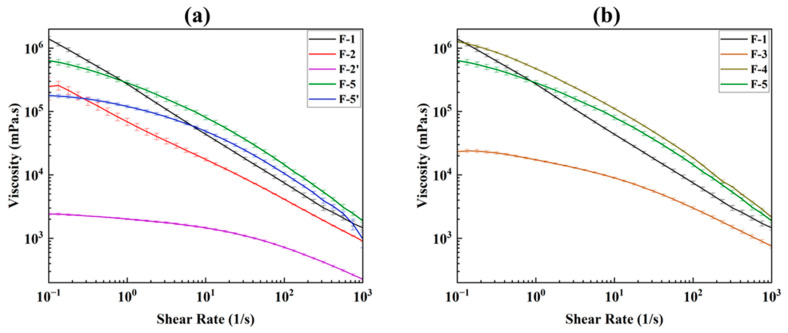
Viscosity profiles comparison for ink formulation (**a**) F-1, F-2, F-2’, F-5, and F-5’ and (**b**) F-1, F-3, F-4, and F-5.

The flow test provides viscosity profiles. The viscosity profile (viscosity vs. shear rate) of an ink is crucial, as inks with low viscosity tend to have poor shape fidelity after 3D printing, while inks with high viscosity can be difficult to extrude [55,56] during 3D printing. Hence, viscosity profiles of all the formulated inks (F-1–F-5’) were determined, compared, and presented in Figure 4a,b. The viscosity profile then correlates with the 3D-printed film (Figure 3). The viscosity profiles of all the inks are presented in Figure 4a,b to minimize overcrowding and make comparisons clearer. Each ink’s viscosity profile is compared with those of F-1 and F-5, as these two inks yielded good 3D-printed film (see Figure 3). Overall, all inks exhibit shear thinning behavior, where the viscosity decreases with increasing the shear rate, which is considered suitable for extrusion-based 3D printing techniques [57,58]. However, not all the inks were successful in the 3D printing of the film (see Figure 3). A detailed analysis is discussed below to understand and correlate this printing outcome with the viscosity profile. 

**Figure 5:**
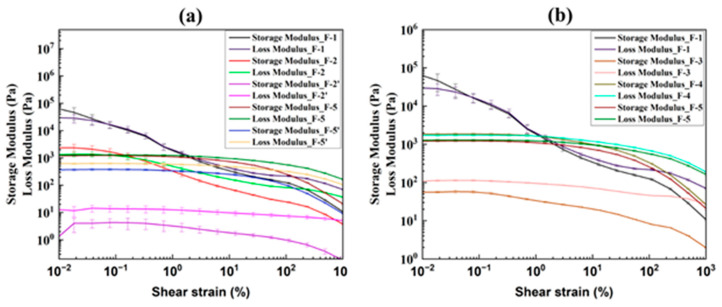
Amplitude sweep curve comparison for ink formulation (**a**) F-1, F-2, F-2’, F-5, and F-5’ and (**b**) F-1, F-3, F-4, and F-5.

A comparison between inks F-1 and F-2 showed that ink F-2 has a lower viscosity than F-1 (Figure 4a). This is due to the presence of low molecular weight glycerin (92.09 g/mol) [59] in F-2 instead of the high molecular weight of PEG 6000 (average molecular weight 5000–7500 g/mol) [60] in F-1. The viscosity of the ink increases as the polymer’s molecular weight increases [61]. The viscosity of the ink F-2’ dropped significantly and was unprintable (Figure 3) due to the removal of mannitol, which caused a subsequent increase of water content from 39.20% *w/w* to 64.30% *w/w*. To overcome this significant drop in viscosity, ink F-3 was prepared with the swellable cross-linked biopolymer SSG that was added to the formulation [34]. The addition of SSG with the presence of SA increased the ink’s viscosity (Figure 4b). This enhanced viscosity in ink F-3 was observed because the SSG particles swelled by absorbing water. The swelling/hydration capacity of SSG is 23.6 cm^3^/g [40,45]. This water absorption leads to an increase in the effective SA concentration and solids (drug and SSG particles) loading by reducing the free volume of the water and limiting the water’s motion, thus accounting for the higher viscosity caused by SSG [34]. The printing outcome shows that the printing of the film was unsuccessful (Figure 3 F-3). Drops of ink were observed, which indicates that the ink viscosity needs to be further increased. Hence, the polymer SA percentage was increased from 0.8 (0.24 × 100/30) % *w/w* to 2.67 (0.80 × 100/30) % *w/w* (ink F-4) instead of increasing SSG concentration (due to its high swelling capacity) to further enhance the ink’s viscosity. However, the resulting ink’s viscosity was too high for printing (Figure 3 F-4 and Figure 4b), so the amount of SSG was reduced from 2 (0.60 × 100/30) % to 1.67 (0.50 × 100/30) % and the ink F-5 was formulated. The viscosity profile of ink F-5 showed the shear-thinning behavior of the ink and was suitable for 3D printing of the film (Figure 3 F-5). The ink F-5’ was prepared to observe the effect of SSG alone, without glycerin, on the viscosity of ink and, later on, the mechanical properties of the printed film. As expected, the ink F-5’s viscosity decreased compared to F-5 due to the removal of glycerin’s contribution to viscosity (glycerin has a higher viscosity than water) [62] as well as an increase in water content from 60.67% to 70.67% (*w/w*). The 3D printing was not successful. A discontinuity in the ink’s extrusion was observed during the printing (Figure 3 F-5’) due to the absence of glycerin [63]. Overall, only F-1 and F-5 were found to be suitable for PAM 3D printing among the seven inks that were prepared.

#### 3.1.2. Amplitude Sweep Tests

Amplitude sweep tests were conducted to assess the viscoelastic properties of the various inks. These tests illustrate the deformation behavior of the inks in the non-destructive deformation range and determine the upper limit of this range [64]. Figure 5 demonstrates the results of the amplitude sweep test performed at a constant 1 Hz frequency, which shows the storage modulus (G′) and loss modulus (G″) plotted against shear strain (%). The storage modulus indicates the ink’s elastic (solid-state) behavior, while the loss modulus indicates its viscous (liquid-state) behavior [65]. At a constant frequency of 1 Hz, inks F-1 and F-2 showed solid-like behavior (Figure 5a) because G′ dominates G″ at low shear strain and before the intersection or cross-over point. Generally, an ink that shows solid-like behavior, G′ dominates G" up to a certain level of shear strain and then intersects. This intersection defines the linear viscoelastic region (LVE region), which indicates the range in which the test can be carried out without destroying the ink’s internal structure [66,67]. Beyond this intersection, both the storage and loss modulus decrease, with G" dominancy, indicating a liquid-like behavior during which the material flows [25,68]. This information is useful for understanding the relationship between extrusion pressure and material flow [58].

It is noted that both inks, F-1 and F-2, contained mannitol and exhibited G′ dominancy over G" until they reached the intersection or crossover points. Both inks showed solid-like behavior before the crossover point and liquid-like behavior after it. However, they also exhibited brittle fracturing behavior confirmed by the sharp dropping of G′ [64]. For ink F-2’, G′ and G″ decrease significantly due to the removal of mannitol from the formulation of the ink (Table 1), and G″ dominates G′ (Figure 5a, F-2’), showing the liquid-like behavior during printing and the inability of the film to hold its structure (Figure 3, F-2’). In ink F-3, SSG was added, which increased the modulus compared to F-2’s with a discrepancy in which G″ dominates G′ (Figure 5b, F-3). The ink F-3 was able to extrude sporadically from the nozzle due to SSG’s liquid-like nature and tendency to flow even at rest (Figure 3, F-3) [69]. The ink F-4’s modulus was further increased due to an increase in SA percentage (0.8% to 2.67%). However, a higher SA concentration results in ink stiffness [70], causes clogging issues, develops gaps during printing, and eventually provides an inconsistent print (Figure 3, F-4). The ink formulation modification from F-4 to F-5 was made by adjusting SSG concentration from 2% to 1.67% (Table 1), resulting in better film printing without any issues (Figure 3, F-5). The ink F-5’ had the proper modulus values (close to the modulus value of F-5). However, there were printing issues (Figure 3, F-5’) due to the absence of the plasticizer, as plasticizer glycerin was removed from the ink [63]. 

#### 3.1.3. Thixotropy Test

**Figure 6:**
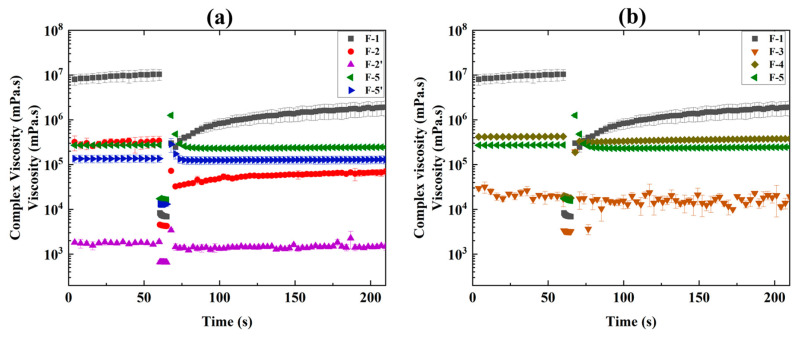
Thixotropy profile of ink formulation (**a**) F-1, F-2, F-2’, F-5, and F-5’ and (**b**) F-1, F-3, F-4, and F-5.

The thixotropy test consists of three intervals and focuses on the ink’s time-dependent structural recovery after shear is applied to assess the post-printing behavior of the ink. It simulates the extrusion behavior of the ink from the printer cartridge [71]. When subjected to high shear rates, the ink exhibited a rapid decrease in viscosity due to its shear-thinning behavior. The ink’s ability to restore and return to its original viscosity after high shear rates indicates the likelihood of the ink spreading in post-printing and measuring the shape fidelity of the printed objects [72]. Figure 6 shows the thixotropy profile (viscosity change with time) of all the formulated inks (F-1 to F-5’). The thixotropy test consists of three intervals: the first interval applies a very low shear on the ink to simulate the ink’s behavior at rest, reflecting low strain within the Linear Viscoelastic (LVE) region, where the initial network structures of the polymer begin to break down [73]. The second interval applies a high shear on the ink to simulate the structural breakdown of the sample during extrusion, representing a high strain far beyond the LVE region. Finally, the third interval applies the same low strain on the ink as the first interval to simulate the ink’s structural recovery at rest. During 3D printing, as the ink is extruded through the nozzle, shear forces disrupt the bond between polymer chains [74,75,76]. Stress is released once the ink is extruded and the internal network begins to recover [74]. However, this recovery takes time, and in most cases, some bonds remain irrecoverable even though the shear is released, resulting in a recovery rate of less than 100% [58]. 

An analysis of the thixotropy profiles for all the inks revealed that formulations F-1 and F-2, having mannitol, exhibited (Figure 6a) slow recovery over time, suggesting that these inks required a prolonged time to regain their initial internal structure. On the contrary, formulations from F-3 to F-5 without mannitol showed quick recovery. Formulations F-2’ and F-3 show fluctuations in recovery due to rapid viscosity changes during the measurement (Figure 6a,b). These two inks also have low viscosity compared to other inks. It is interesting to note that this study found both slow (ink F-1) and quick (ink F-5) recovery rates were suitable for printing in terms of shape fidelity based on visual observation (Figure 3). A closer look at Table 1 shows that the difference between these two inks is the excipients mannitol vs. SSG and the change in SA concentrations (0.8% vs. 2.67%). A comparison between F-2 and F-2’ shows that mannitol impacted slow recovery, as the only difference between these two inks is the presence of mannitol. On the other hand, a comparison between F-3 and F-4 shows both inks had a quick recovery, though there is a difference in SA concentrations (0.8% vs. 2.67%). The variation in SA concentrations does not impact the recovery. Hence, it is reasonable to say mannitol is responsible for the slow recovery, whereas SSG helps to quick recovery. The rest of the inks (F-4 and F-5’) that had SSG (Table 1) showed quick recovery, which confirms this finding. Generally, inks with low recovery percentages take a longer time to regain their structure and are prone to spreading during post-printing, leading to poor shape fidelity [72]. However, here, both inks, F-1 and F-5, displayed different recovery (slow vs. quick) patterns (Figure 6a), and both produced visually satisfactory films (Figure 3). The difference between these films was observed in their mechanical properties, which are discussed in the following section.

### 3.2. Mechanical Properties of Film

Films must possess adequate strength and flexibility to avoid tearing during the cutting, packaging stages, and transportation [49]. Generally, Young’s Modulus (YM), Tensile Strength (TS), and Elongation at Break (EB) are measured to evaluate the film’s mechanical properties. The mechanical characteristics of polymer films depend on their composition, including the type and amount of polymer and plasticizer used [49]. If a film is too stiff, as indicated by a high YM, it may cause discomfort in the mouth [49]. Therefore, it is crucial to have a flexible and mechanically robust film. TS evaluates a film’s strength, while EB determines its toughness and capacity to stretch before breaking. The YM, TS, and EB of the films (F-1, F-5, and F-5’) have been analyzed, and the findings of these evaluations are presented in Table 2. It is noted that only F-1, F-5, and F-5’ inks were successfully 3D printed (see Figure 3). Hence, only their mechanical properties were evaluated. The films developed using the F-1 and F-5’ inks did not exhibit desirable mechanical properties, whereas the films developed using F-5 ink demonstrated good mechanical properties (Table 2). The F-1 film, which contained mannitol and PEG, could not undergo mechanical testing due to its brittleness. The F-5’ film that was composed of SSG broke during mechanical testing, making it impossible to measure its mechanical properties. In contrast, the F-5 film, which included SSG and the plasticizer glycerin, showed good mechanical properties within the range reported in the literature [44,49]. It is noted that since F-1 and F-5’ films could not provide mechanical testing data, a picture of brittle film was shown here. On the other hand, F-5 films’ mechanical testing data were presented, and the picture was not considered. Glycerin is a widely used plasticizer in pharmaceutical formulations. Plasticizers play a vital role in improving the flexibility of biopolymer-based films [77] by reducing the internal hydrogen bonds between polymer chains, thus increasing molecular spacing in the film and enhancing the film’s flexibility [78]. As a small molecule, glycerin quickly penetrates between polymer chains, influencing the properties of the film. The findings indicated that SSG alone could not enhance the film’s mechanical properties. Incorporating glycerin as a plasticizing agent enhances flexibility and mitigates the film’s brittleness. 

### 3.3. Fenofibrate Particles Crystallinity in Film 

**Figure 7:**
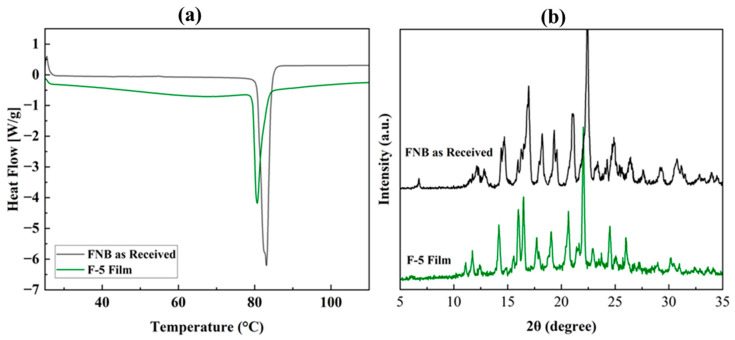
(**a**) DSC thermograms of FNB drug (fenofibrate as-received) and F-5 film and (**b**) XRD diffractograms of FNB drug (fenofibrate as-received) and F-5 film.

The differential scanning calorimeter (DSC) and X-ray diffraction (XRD) analyses of the film (F-5) and the as-received FNB drug were performed to evaluate FNB crystallinity in the film. A DSC was used to determine the crystallinity and phase transitions (if any) of the FNB drug embedded in the film compared with the as-received FNB powder [79]. The DSC thermogram of FNB (as-received) and dried film is presented in Figure 7a. The phase transition sharp peaks were the melting temperatures of the FNB (as-received) and the embedded FNB in the film. The melting temperature for as-received FNB is 80–81 °C, while the melting temperature of the embedded FNB in film ranges from 80 °C to 82 °C [80]. The DSC results suggested that the embedded FNB in the film did not exhibit any polymorphic phase, and the crystalline nature of FNB was preserved after film formation. Significant crystal defects would result in a substantial reduction in melting temperature, which was not observed. In addition, the embedded FNB peak did not shift compared to the as-received FNB, implying that the embedded FNB did not interact with the film polymers and other additives [79,81]. This further validated the crystalline nature of FNB in film. However, the reduced endothermic peak observed for the film might be due to the dilution effect. Besides the DSC, an XRD analysis of the dried 3D thin film (F-5) was conducted to support whether FNB maintained its crystalline structure after printing. Figure 7b shows the XRD pattern for the as-received FNB powder and the printed F-5 film. The XRD patterns are very similar, indicating that the FNB crystalline structure remains unchanged in the film even after printing and subsequent drying.

### 3.4. Analysis of FNB Particle Distribution in Films 

**Figure 8:**
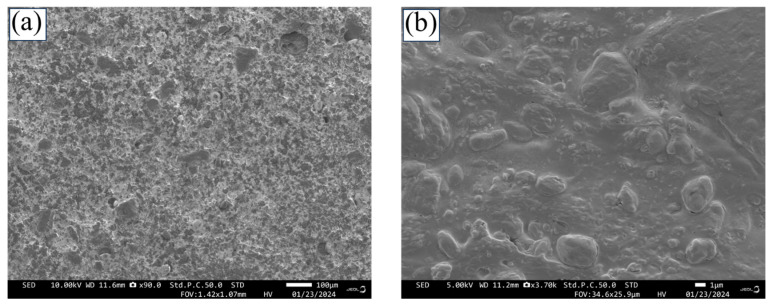
SEM images of F5 film’s surface at (**a**) 90× magnification and (**b**) 3700× magnification.

The 3D-printed film morphology and FNB particle distribution within the film (F-5) were qualitatively assessed through scanning electron microscopy (SEM) imaging. At a lower magnification (90×), Figure 8a demonstrates that the film is free of cracks and shows the presence of particles, including the FNB drug, across the film. However, it should be noted that the encapsulation of FNB particles within the polymer obscures their visibility in SEM images. Hence, a higher magnification image provides better visibility and was taken. At higher magnification (3700×), Figure 8b shows that polymer-embedded particles (FNB and SSG) were scattered across the film. The FNB and SSG particles are distributed well in the printed film, though they cannot be as distinguished on SEM images. 

### 3.5. Drug Loading and Dissolution of FNB Particles from Films

**Figure 9:**
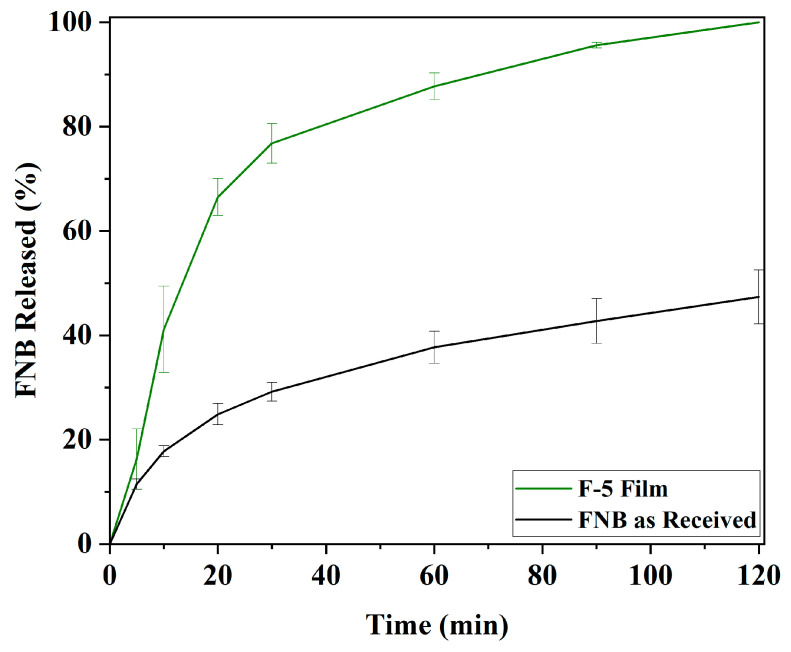
Dissolution profile for the F-5 film containing FNB drug particles and as-received FNB particles in SDS media.

As described in Section 2.6.5, all six films from ink formulation F-5 were analyzed for drug loading, and the results are provided in Table S1. The average drug loading in the films was 44.28% (*w/w*), with a relative standard deviation (RSD) of 5.62%. The findings suggest that FNB is evenly distributed across the six films printed using the F-5 ink [82]. Dissolution tests were conducted using the basket method to determine the FNB release profile of the F-5 films. The dissolution profiles of F-5 film and as-received FNB are presented in Figure 9. A distinct difference is observed between the two profiles, confirming that FNB dissolves faster in the film than the as-received FNB powder. A complete drug release from the films was achieved within 120 min, whereas, as received, FNB powder dissolved only 47%. A closer look at the F-5 film dissolution profile shows more than 77% dissolve within 30 min. To investigate such a fast release, it is found that the dried film contains SA, PVP, and SSG, where SSG acts as a super disintegrant that aids in the rapid disintegration of the film [82]. SSG has a quick moisture absorption property, resulting in swelling and subsequent expedited breakdown of pharmaceutical dosage; in this case, it is the film [83]. Hence, a faster drug release from the films is likely due to their rapid breakdown or disintegration, which generates more pores and channels, facilitating the drug’s diffusion out of the films [84].

It is noted that the as-received FNB powder was not fully dissolved in 2 h even though sink conditions were maintained throughout the dissolution test. A complete dissolution of as-received FNB powder was not achieved for several reasons: (a) particle size and surface area: larger drug particles or those with a lower surface area may dissolve more slowly, preventing complete dissolution within the test timeframe [85]; (b) diffusion layer limitations: as the drug dissolves, a stagnant layer of saturated solution can form around the particles, slowing further dissolution despite overall sink conditions in the bulk medium [86]; (c) agglomeration: particles form agglomerates that reduce the effective surface area and slow dissolution [87].

## 4. Conclusions

The swellable cross-linked biopolymer SSG was successfully incorporated into an SA-based polymer ink to use in the 3D printing of films. Including SSG effectively enhanced the ink’s rheological properties, such as exhibiting shear-thinning behavior with suitable viscosity, higher modulus, and quick recovery, leading to suitable 3D printing. The PAM-type 3D printer operated at a speed of 10 mm/s, printing pressure of 60 kPa, and an infill density of 50% was able to successfully fabricate FNB-embedded thin film with dimensions (15 mm × 10 mm × 0.35 mm). It was found that SSG alone cannot improve the film’s mechanical properties, which confirms that a plasticizer is essential. Adding glycerin as a plasticizer in the ink formulation improved the mechanical properties, resulting in a desirable Young’s modulus (23.67 kPa), tensile strength (329.67 kPa), and elongation at break (12.39%). The film had a drug loading of 44.28% with very good uniformity (RSD 5.62%). SEM analysis of the films revealed particle (FNB, SSG) distribution throughout the film. FNB retained its crystallinity in the dried film, as confirmed by XRD and DSC analyses. The 3D-printed film exhibited a superior FNB drug dissolution profile compared to the as-received FNB. Overall, the study of ink rheology and the performance of FNB-incorporated film demonstrated that the swellable cross-linked biopolymer SSG provides enhanced ink rheology, is suitable for PAM 3D printing, and provides better drug dissolution. Glycerin as a plasticizer is essential with SSG to achieve the desired mechanical properties of the 3D-printed film.

## Figures and Tables

**Table 1:** The formulation used for preparing ink.

Formulation ID	SA (Polymer) (g)	FNB (Drug) (g)	Mannitol (Filler) (g)	SSG (Cross-Inked Biopolymer) (g)	PVP (Stabilizer) (g)	PEG or Glycerin * (Plasticizer) (g)	Water (Solvent) (g)	Total (g)
F-1	0.24	6	7.50	-	1.50	3	11.76	30
F-2	0.24	6	7.50	-	1.50	3	11.76	30
F-2’	0.24	6	-	-	1.50	3	19.29	30
F-3	0.24	6	-	0.60	1.50	3	18.66	30
F-4	0.80	6	-	0.60	1.50	3	18.10	30
F-5	0.80	6	-	0.50	1.50	3	18.20	30
F-5’	0.80	6	-	0.50	1.50	-	21.20	30

* Only formulation F-1 contains PEG. All other formulations contain glycerin.

**Table 2:** Mechanical properties of thin film.

Formulation ID	Young’s Modulus (YM), (kPa)	Tensile Strength (TS), (kPa)	Elongation at Break (EB), (%)	Observation	
**F-1**	-	-	-	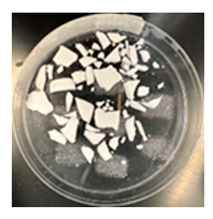	Brittle film
F-5	23.67 ± 7.48	329.67 ± 26.58	12.39 ± 2.54	-	Has sufficient mechanical strength
F-5’	-	-	-	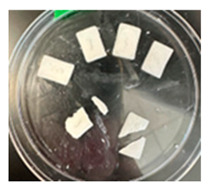	Brittle film

## Data Availability

Additional data will be made available on request.

## Supplementary Materials

The following supporting information can be downloaded at https://www.mdpi.com/article/10.3390/pharmaceutics17020183/s1, Table S1: F-5 formulation printed film’s drug loading. Figure S1: Calibration curve for fenofibrate concentration measured at 286 nm.
