# Supplementary material for: Evaluating Swellable Cross-Linked Biopolymer Impact on Ink Rheology and Mechanical Properties of Drug-Contained 3D-Printed Thin Film
Source: Pharmaceutics. 2025 Feb 1;17(2):183. doi: 10.3390/pharmaceutics17020183 (PMC11858972; doi:10.3390/pharmaceutics17020183)
Supplement: Supplementary file 1 [file pharmaceutics-17-00183-s001.zip › pharmaceutics-3391056-supplementary.pdf]

Article

Evaluating Swellable Cross-Linked Biopolymer Impact on Ink Rheology and Mechanical Properties of Drug-Contained 3D-Printed Thin Film

Farzana Khan Rony [1,2], Jonathan Appiah [2], Asmaa Alawbali [2], Distinee Clay [2], Shamsuddin Ilias [3], and Mohammad A. Azad [2,*]

[1] Department of Applied Science and Technology, North Carolina A&T State University, Greensboro, NC 27409, USA; fkrony@aggies.ncat.edu
[2] Materials Science and Process Engineering (MSPE) Lab, Department of Chemical, Biological, and Bioengineering, North Carolina A&T State University, Greensboro, NC 27409, USA; jappiah@aggies.ncat.edu (J.A.); djclay@aggies.ncat.edu (D.C.)
[3] Department of Chemical, Biological, and Bioengineering, North Carolina A&T State University, Greensboro, NC 27409, USA; ilias@ncat.edu
* Correspondence: maazad@ncat.edu; Tel.: +1 -336-285-3701; Fax: +1 -336-334-7904

Table S1. F-5 formulation printed film's drug loading.

Film	Film weight (mg)	Drug loading in a film	
		(mg)	(%)
1	43.70	21.26	48.64
2	46.00	20.53	44.64
3	47.40	19.40	40.94
4	50.30	21.02	41.80
5	37.30	16.71	44.81
6	38.50	17.26	44.84
		Average	44.28
		STDEV	2.49
		RSD	5.62

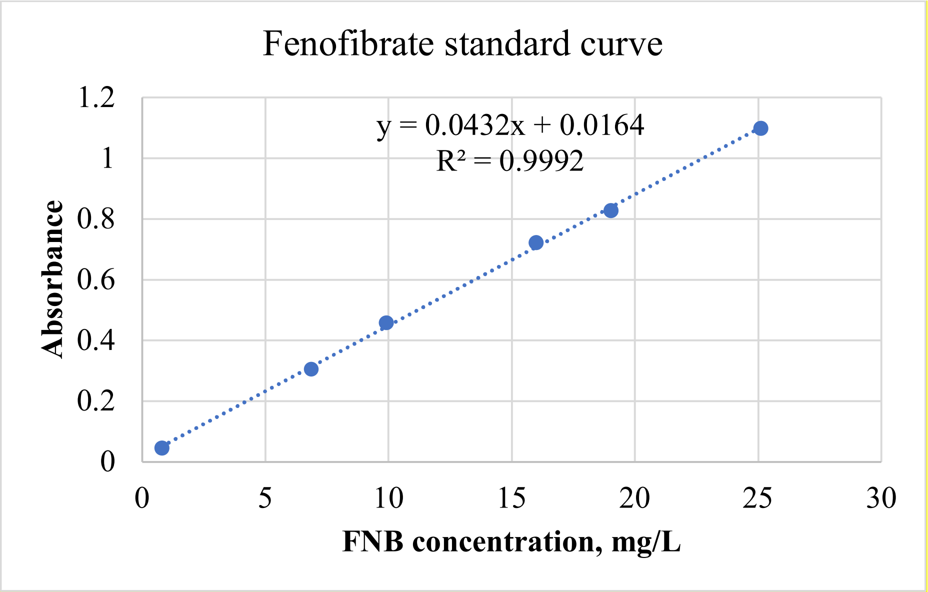

Figure S1: Calibration curve for fenofibrate concentration measured at 286 nm.
